# Identification hub genes of consensus molecular subtype correlation with immune infiltration and predict prognosis in gastric cancer

**DOI:** 10.1007/s12672-021-00434-5

**Published:** 2021-10-16

**Authors:** Xin Yu, Bin Yu, Weidan Fang, Jianping Xiong, Mei Ma

**Affiliations:** 1 Department of Oncology, The First Affiliated Hospital of Nanchang University, Nanchang, 330006 Jiangxi China; 2 Department of General Surgery, The First Affiliated Hospital of Nanchang University, Nanchang, 330006 Jiangxi China

**Keywords:** Gastric cancer, Prognosis, Immune infiltration, OGN, CHRDL2

## Abstract

Gastric cancer (GC) has a great fatality rate, meanwhile, there is still a lack of available biomarkers for prognosis. The goal of the research was to discover key and novel potential biomarkers for GC. We screened for the expression of significantly altered genes based on survival rates from two consensus molecular subtypes (CMS) of GC. Subsequently, functional enrichment analysis showed these genes involved in many cancers. And we picked 6 hub genes that could both secreted in the tumor microenvironment and expression enhanced in immune cells. Then, Kaplan Meier survival and expression detected in the tumor pathological stage were utilized to clarify the prognostic of these 6 hub genes. The results indicated that OGN, CHRDL2, C2orf40, THBS4, CHRDL1, and ANGPTL1, respectively, were significantly associated with poor OS in GC patients. And their expression increased with cancer advanced. Moreover, immune infiltration analysis displayed that those hub genes expression positively with M2 macrophage, CD8+ T Cell, most immune inhibitors, and majority immunostimulators. In summary, our results suggested that OGN, CHRDL2, C2orf40, THBS4, CHRDL1, and ANGPTL1 were all potential biomarkers for GC prognosis and might also be potential therapeutic targets for GC.

**Supplementary Information:**

The online version contains supplementary material available at 10.1007/s12672-021-00434-5.

## Introduction

Gastric cancer (GC) is one of the most common malignant tumors, and its mortality ranks fourth among all malignant tumors in the world [1]. According to the global cancer statistics (2020), there were over one million new cases of GC and with a mortality rate of 7.7% of the total cancer deaths [1]. Due to the lack of effective biomarkers, most patients with GC are diagnosed at an advanced stage and distant metastasis often occurs at this stage, leading to a poor prognosis. The 5-year overall survival rate of GC is about 20–30% in most parts of the world [2]. The earlier the effective intervention for GC patients, the more beneficial to improve their survival rate. Therefore, it is necessary to identify the key prognostic biomarkers in early time for GC therapy.

Immune checkpoint blockade (ICB) has been brought new hope for many cancers treatment. While ICB does not valid in most cases, including GC [3], and thus more research is required to drive the field forward. Immune-related cell in the tumor microenvironment (TME) is also an indispensable factor for the development, progression, and prognosis of GC. Despite CD8+ T cells are considered to be an anti-tumor component in the TME [4], studies on the relationship between CD8+ T cells and prognosis in GC yielded in divergence [5, 6]. Programmed cell death protein 1 (PD-1) is an important immune checkpoint molecule in CD8+ T cell and its upregulation disturbs the anti-tumor function of CD8+ T cell [7]. PD-1+CD8+ T cell infiltration was significantly increased in patients with GC [6]. While PD-1 blockade combined with immune modulators, such as TGF-β1 inhibitor, might take patients more benefits than targeted PD-1 alone [6]. And it was also reported that some subsets of CD8+ T, such as CXCR5+CD8+ T [8], but not CD8+ T itself connected with better overall survival (OS) in patients with GC. Therefore, it is important to find out the key molecules that regulate immune modulators in TME or/and co-express molecular in CD8+ T that mediated its function.

Besides, the latest studies have proved that macrophages, mostly M2 polarized macrophages, have a vital role in the GC advance [9, 10]. Infiltration of M2 macrophage might be a worthwhile biomarker for GC prognosis [11]. Targeting M2 macrophages may be a promising therapeutic strategy, therefore, it is valuable that uncover M2-related molecules.

Here, we picked out those genes that were significantly associated with the survival of GC patients. And selected hub genes, OGN, CHRDL2, C2orf40, THBS4, CHRDL1, and ANGPTL1, which secreted into TME and enhanced in immune cells. After validated their expression value for GC diagnosis, we performed immune infiltration analysis of these 6 hub genes. Our study might provide novel biomarkers for GC advance, and potential targets that combined using with ICB to improve GC therapy.

## Results

### Identification of robust genes associated with GC survival

**Fig. 1:**
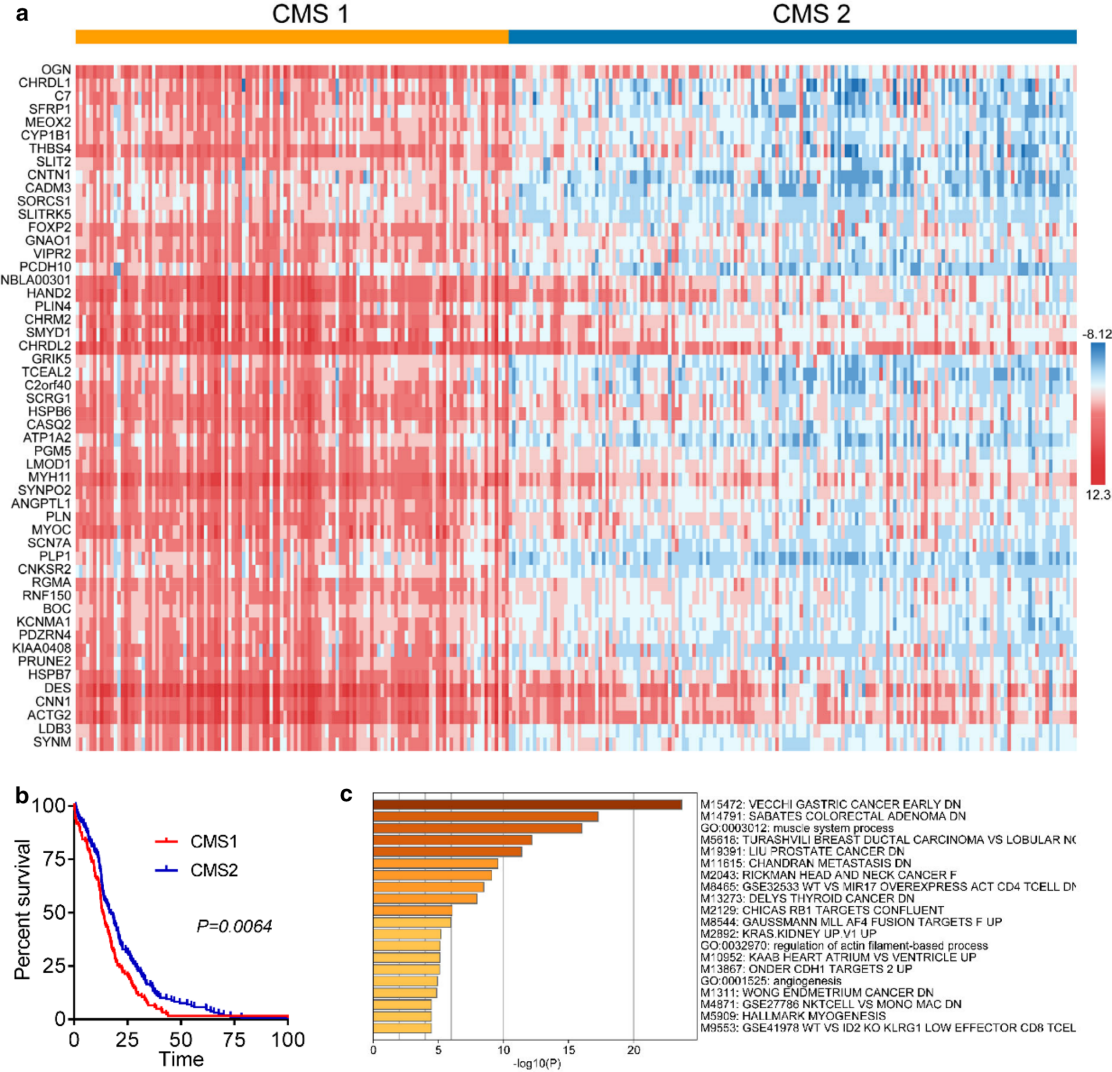
Identification of robust genes associated with GC survival and their functional enrichment analysis. **a** Identification of key modules that prominent change in CMS1 vs. CMS2.** b** Kaplan–Meier survival analysis showed CMS1 had poor survival. **c** Gene ontology (GO) enrichment analysis of CMS selected genes in Metascape

The COMSUC web server [12] was used to find out the hub genes which significantly correlated with the survival of GC patients. We firstly clustered GC samples into two consensus molecular subtypes (CMS) by multiple methods, including K-means, hierarchical clustering (Hclust), non-negative matrix factorization (NMF) (Supplementary Fig. S1a, b). Among them, we found that 52 genes prominently up-regulated expression in CMS1 compare to CMS2 (Fig. 1a). And CMS1 had lower survival than CMS2 group (Fig. 1b). These genes may act independently or work in concert for GC survival.

### Functional enrichment analysis of survival-related CMS genes

To identify the notably enriched pathway and biological process of survival-related CMS genes, we explored in Metascape website [13] and found those genes related to many processes of cancer (Fig. 1c). And the most relative pathway was early gastric cancer (Fig. 1c). Then we analyzed the sub-network of those CMS1 genes in TCGA RNAseq of STAD, most of them had some connections in each other (Supplementary Fig. S1c). According to those results, we speculated whether those genes were regulated by some common transcription factors (TFs). After performing TF enrichment in Knock TF (http://www.licpathway.net/KnockTF/index.html), more than ten TFs predicted may regulate over 73% of genes of these selected 52 genes (Table 1). The top 4 TFs were TFAP4, TP53, CREB1, and PTEN, respectively (Supplementary Fig. S1d), and they all have been reported to promote the progression of GC [14–17]. Furthermore, we explored the characteristics of the 52 genes in the GeneCards (https://www.genecards.org/) and the Human Protein Atlas (https://www.proteinatlas.org/). As shown in Table 2, 10 genes could be transcribed into secreted proteins, 22 genes enriched in immune cells and only 7 genes own both characters (Supplementary Fig. S1e).

**Table 1:** Selected 52 genes with CMS1-related significant change performed TF enrichment in Knock TF

TFs	Genes regulated by relative TFs
TFAP4	LDB3 KIAA0408 SLITRK5 SORCS1 VIPR2 SYNMPRUNE2 ACTG2 GNAO1 RGMA HSPB7 CASQ2 DES MYH11 BOC SYNPO2 MYOC GRIK5 CHRM2 PGM5 LMOD1 SMYD1 THBS4 CADM3 CHRDL2 SCRG1 FOXP2 SCN7A ATP1A2 HAND2 PDZRN4 TCEAL2 HSPB6 PLIN4 CNKSR2 RNF150 CYP1B1 SFRP1 CNN1 CHRDL1 OGN C7 SLIT2 PLNvMEOX2 C2orf40 ANGPTL1 PLP1 CNTN1 KCNMA1 PCDH10
TP53	MEOX2 SLITRK5 PLP1 KIAA0408 CYP1B1 CHRDL1 FOXP2 CASQ2 PCDH10 HAND2 RGMA HSPB6 MYH11 CNN1 CNTN1 SFRP1 KCNMA1 OGN SCN7A MYOC ATP1A2 SCRG1 HSPB7 ACTG2 SMYD1 DES LDB3 CHRDL2 GNAO1 THBS4 GRIK5 CHRM2 SLIT2 ANGPTL1 CNKSR2 CADM3 BOC C2orf40 SYNPO2 LMOD1 PGM5 RNF150 PLN VIPR2 PLIN4 TCEAL2 PDZRN4 SORCS1
CREB1	CASQ2 SMYD1 CHRM2 PGM5 TCEAL2 MEOX2 PLP1 C7 KIAA0408 SYNPO2 DES KCNMA1 LDB3 PLIN4 MYOC GNAO1 SCRG1 FOXP2 SLIT2 SCN7A CYP1B1 ANGPTL1 PCDH10 ATP1A2 PRUNE2 SLITRK5 CNN1 CNTN1 ACTG2 LMOD1 C2orf40 PLN CADM3 SORCS1 OGN THBS4 CHRDL2 RGMA HSPB7 SFRP1 CHRDL1 GRIK5 HAND2 HSPB6 PDZRN4 CNKSR2
PTEN	KIAA0408 PCDH10 CHRDL1 GNAO1 CNTN1 SLITRK5 SLIT2 SMYD1 SYNM SCRG1 BOC GRIK5 HSPB6 LMOD1 LDB3 MEOX2 TCEAL2 CNN1 C2orf40 ANGPTL1 SCN7A CYP1B1 PLP1 HAND2 PLIN4 THBS4 CASQ2 C7 CADM3 RGMA CHRM2 PDZRN4 SFRP1 OGN PGM5 RNF150 SORCS1 HSPB7 SYNPO2 VIPR2 DES PLN ACTG2 CHRDL2 CNKSR2 ATP1A2
GATA2	CHRDL1 ACTG2 CNN1 SFRP1 KIAA0408 SCRG1 PLIN4 CASQ2 HAND2 PLP1 ATP1A2 SYNM GRIK5 CADM3 THBS4 MYH11 SLIT2 MYOC ANGPTL1 SCN7A KCNMA1 RNF150 GNAO1 LMOD1 PCDH10 MEOX2 CHRM2 TCEAL2 CHRDL2 LDB3 OGN PRUNE2 RGMA CNKSR2 CYP1B1 HSPB7 FOXP2 SMYD1 PLN PDZRN4 SLITRK5 SYNPO2 PGM5 HSPB6 DES
TP63	CYP1B1 PLN SLITRK5 OGN SCN7A KCNMA1 C2orf40 GNAO1 PCDH10 CNKSR2 FOXP2 LMOD1 MYH11 SLIT2 PGM5 C7 HSPB6 LDB3 ANGPTL1 SFRP1 CNTN1 SORCS1 HAND2 CNN1 SYNM PDZRN4 CHRM2 PLIN4 MEOX2 KIAA0408 THBS4 TCEAL2 ACTG2 PLP1 HSPB7 VIPR2 CHRDL1 SCRG1 PRUNE2 GRIK5 BOC RNF150 CHRDL2
FOXM1	CNKSR2 KCNMA1 SORCS1 PLP1 ATP1A2 KIAA0408 CASQ2 CYP1B1 HSPB7 PRUNE2 ACTG2 HAND2 TCEAL2 DES SCRG1 CHRDL2 PLIN4 GRIK5 C7 MEOX2 LDB3 SMYD1 SFRP1 CNN1 MYOC SLIT2 OGN MYH11 SCN7A CADM3 PDZRN4 RNF150 LMOD1 PCDH10 RGMA CNTN1 C2orf40 PGM5 SYNM BOC HSPB6 ANGPTL1
FOXP1	CYP1B1 CNN1 SORCS1 PLN LMOD1 HSPB6 CHRDL2 C2orf40 MEOX2 TCEAL2 PGM5 PDZRN4 ANGPTL1 CHRM2 RGMA RNF150 DES PLP1 HSPB7 SCN7A C7 PLIN4 VIPR2 CNKSR2 CHRDL1 CASQ2 HAND2 SLITRK5 SFRP1 OGN CADM3 PRUNE2 CNTN1 KIAA0408 MYOC LDB3 SYNM KCNMA1 SLIT2 GRIK5 GNAO1 SYNPO2
NR2F2	HSPB7 GNAO1 DES KCNMA1 TCEAL2 CNTN1 SORCS1 CHRDL2 CYP1B1 SCRG1 SMYD1 PLP1 SFRP1 MYH11 LDB3 CNN1 C2orf40 CHRM2 ACTG2 BOC PRUNE2 PCDH10 SLIT2 CADM3 MYOC OGN RGMA THBS4 PDZRN4 CNKSR2 PLIN4 HAND2 KIAA0408 CHRDL1 GRIK5 SLITRK5 MEOX2 C7 PGM5 SYNM ATP1A2
POSTN	KIAA0408 ANGPTL1 CNN1 CHRM2 C7 CASQ2 FOXP2 OGN ATP1A2 GNAO1 KCNMA1 SYNPO2 THBS4 HAND2 CADM3 LMOD1 CHRDL1 PLP1 ACTG2 DES CNTN1 VIPR2 GRIK5 C2orf40 SCN7A RGMA SLIT2 RNF150 HSPB7 SFRP1 CNKSR2 LDB3 MYH11 PLN SLITRK5 CHRDL2 MEOX2 SYNM

**Table 2:** Characteristics of the 52 genes that can be transcribed into secreted proteins or enriched in immune cells

Characters	Selected genes
Secreted	C7 THBS4 CHRDL1 SFRP1 SLIT2 CHRDL2 SCRG1 OGN C2orf40 ANGPTL1
Immune cell-enriched	OGN CHRDL1 PLP1 VIPR2 CHRDL2 PGM5 BOC SORCS1 THBS4 GRIK5 CASQ2 PLIN4 TCEAL2 C2orf40 PLN ANGPTL1 SLIT2 GNAO1 SCN7A NBLA00301 MEOX2 CHRM2 CNKSR2

### Survival outcomes in selected genes

**Fig. 2:**
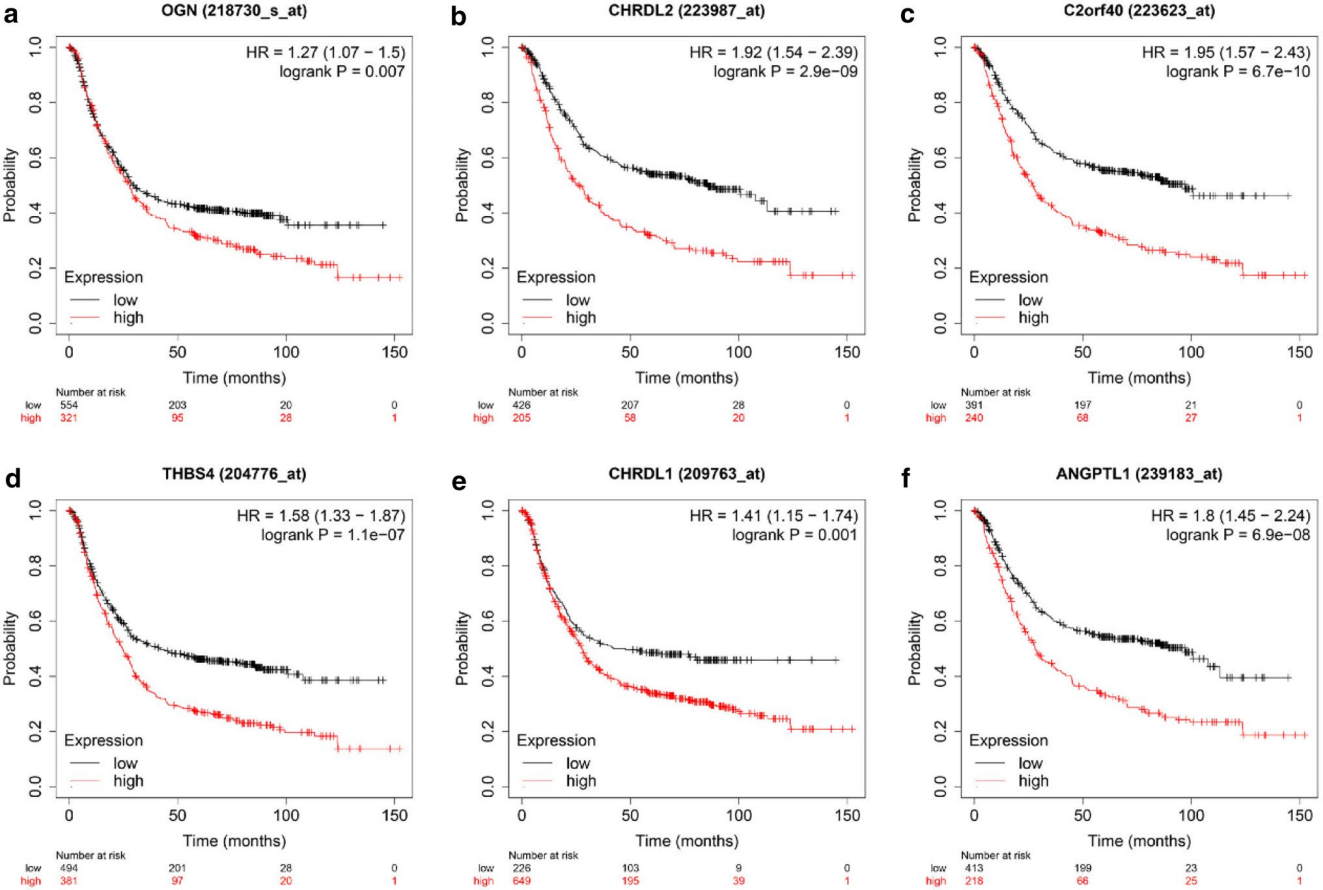
Overall survival analysis of six hub genes in GC patients. Survival curves for high and low expression of OGN, CHRDL2, C2orf40, THBS4, CHRDL1, and ANGPTL1 in GC based on the Kaplan–Meier plotter database

**Fig. 3:**
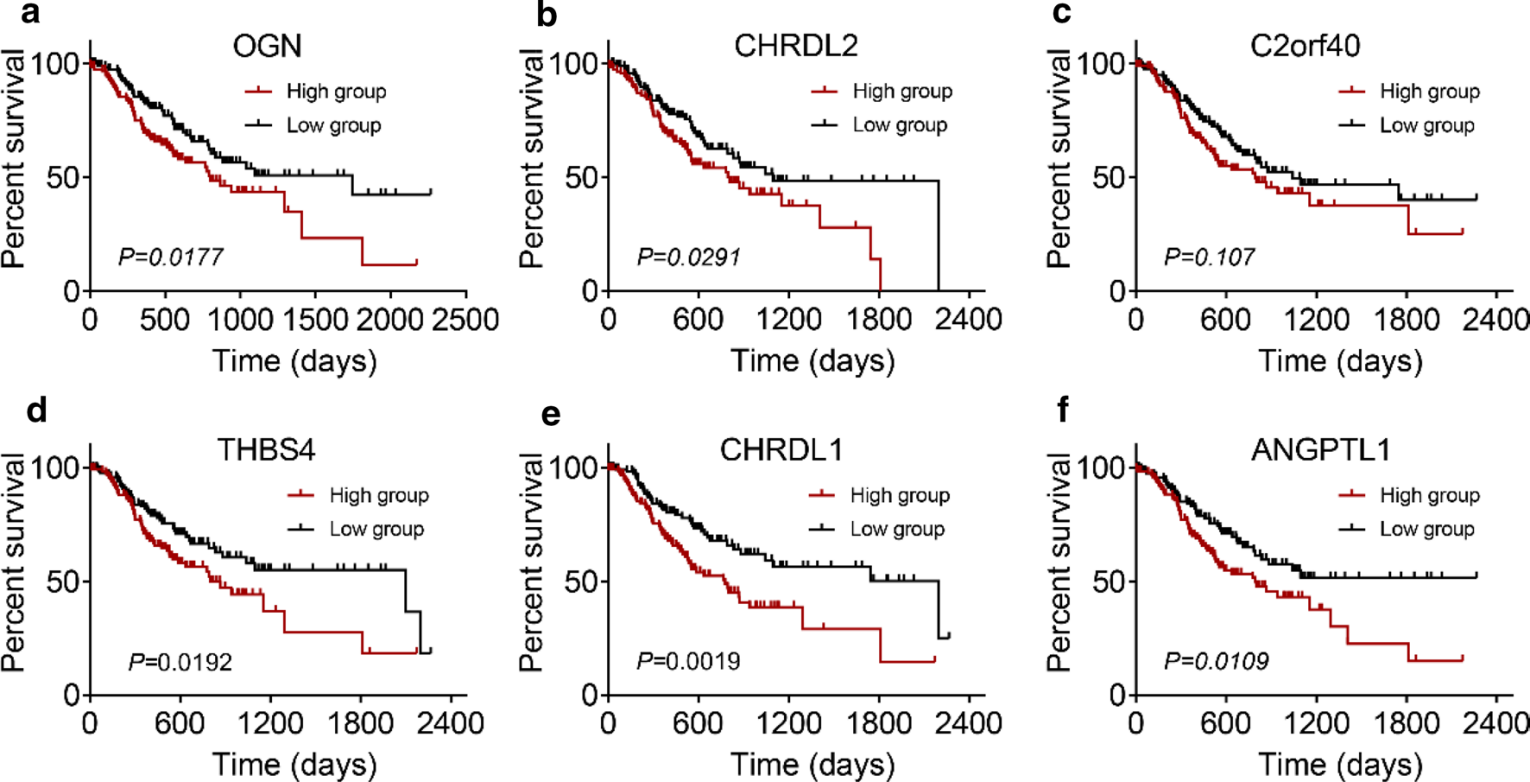
Survival prognosis of six hub genes in high- and low- groups in GC patients using OncoLnc datasets. Kaplan–Meier survival analysis according to the expression of OGN (**a**), CHRDL2 (**b**), C2orf40 (**c**), THBS4 (**d**), CHRDL1 (**e**), and ANGPTL1 (**f**), respectively, were significant differences in GC patients

To verify whether the selected 7 genes (OGN, CHRDL2, C2orf40, THBS4, CHRDL1, ANGPTL1, SLIT2) were correlated with worse overall survival (OS) in GC patients, survival analysis was performed in Kaplan Meier plotter [18]. On account of many articles that have been shown that SLIT2 was over-expression in GC and might be an independent risk factor for GC [19, 20], we did not explore it further in this paper. The Kaplan Meier plotter results showed that the up-regulated expression of OGN, CHRDL2, C2orf40, THBS4, CHRDL1, and ANGPTL1, respectively, was correlated with poor OS in GC patients (Fig. 2a–f). We then observed the prognostic value of these six hub genes in OncoLnc websites [21] and got similar results (Fig. 3a–f). Among them, OGN and CHRDL2 were firstly reported in GC that their expression was related to OS of GC patients. To further confirm the correlation of 6 genes expression with tumor progression in GC, expression in different stages was executed in GEPIA [22]. The analyzed results indicated that the expression of OGN, CHRDL2, C2orf40, THBS4, CHRDL1, and ANGPTL1 was gradually increased in the GC stage (Supplementary Fig. S2a–f). These results revealed these 6 hub genes were significantly related to the aggravation and survival of GC.

### Immune cell infiltration analysis

**Fig. 4:**
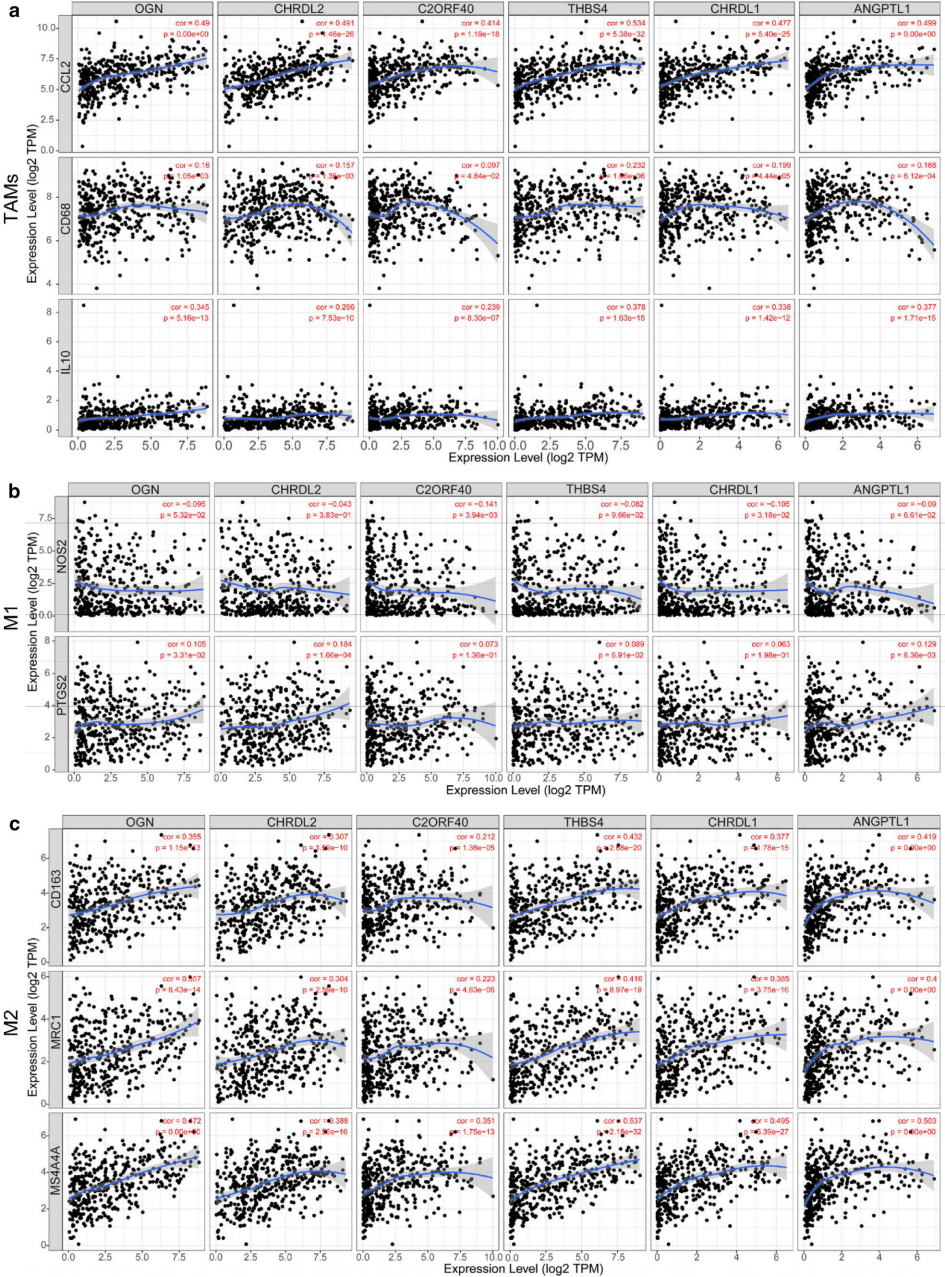
Expression of six hub genes correlated with TAM polarization in the TME of GC. Scatterplots outline the relationship between the expression of six hub genes and various gene markers of TAMs (**a**), M1 macrophages (**b**), and M2 macrophages (**c**) by TIMER

**Fig. 5:**
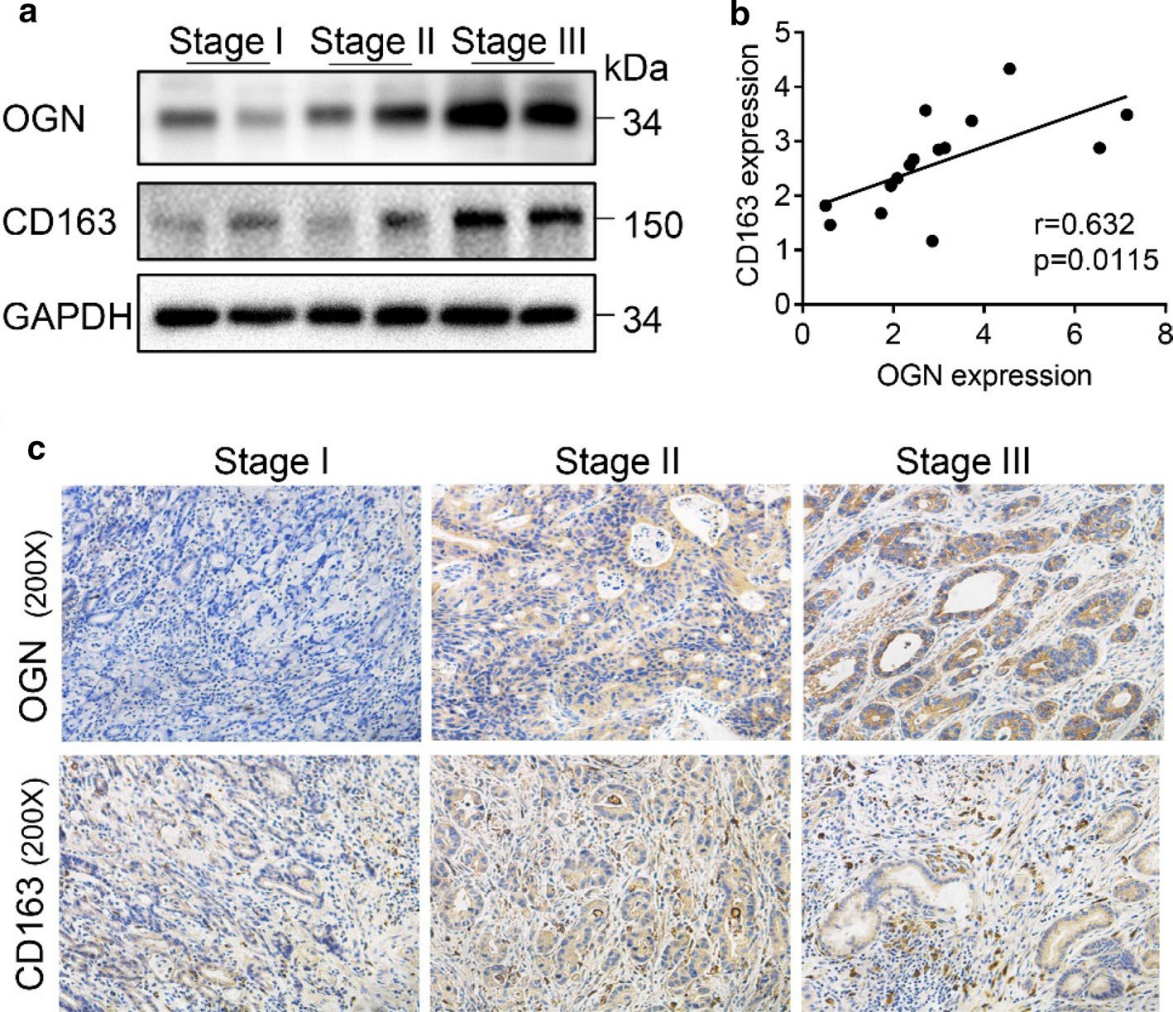
Expression of OGN and its relationship with M2 macrophage in different stages of GC. **a** Western blot showed the expression of OGN and CD163 (marker of M2 macrophage) in different stages of GC. **b** Correlation analysis verified OGN was positively related with CD163 in GC. **c** Representative images of OGN and CD163 in different stages of GC detected by immunohistochemistry staining

To investigate the relationship between the expression of these hub genes (OGN, CHRDL2, C2orf40, THBS4, CHRDL1, and ANGPTL1) and immune infiltration, the TIMER website [23] was used to analyze the correlation between these hub genes and multiple immune cells. The results indicated that the expression of these hub genes was significantly positively correlated with CD8+ T cells, CD4+ T cells, macrophages, neutrophils, dendritic cells (Supplementary Fig. S3). Next, we further analyzed the correlations of macrophage subtypes infiltration with these hub genes in GC. The results exhibited these 6 hub genes had no significant link in M1-related markers with their expression, but exhibited remarkable connection in M2 and TAMs (Fig. 4a–c). We then picked OGN to experimentally validate its expression and its relationship with M2 macrophage. The expression of OGN was increased with GC advance (Fig. 5a), which was consistent with its mRNA expression level showed in GEPIA (Supplementary Fig. S2a). Moreover, our results showed OGN expression correlated with infiltrating of M2 macrophage in different stages of GC (Fig. 5a–c). The above results indicated that these 6 hub genes were associated with immune cell infiltration, while the relationship still needs more evidence to prove.

### Correlation analysis between hub genes and immune markers

**Fig. 6:**
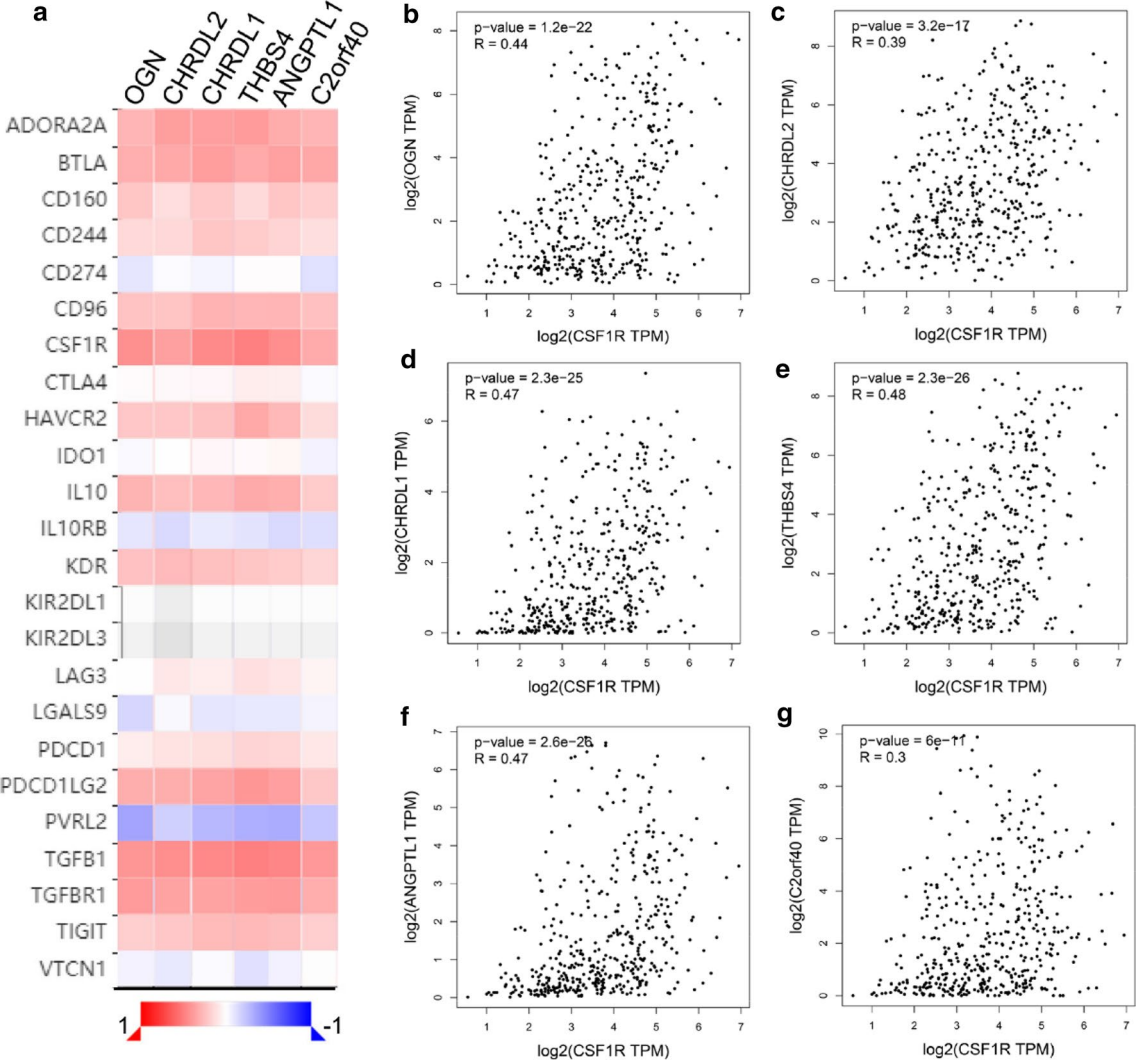
Relations between the abundance of tumor-infiltrating immunoinhibitors and expression of six hub genes. **a** Heatmap exhibited six hub genes that were positively correlated with most immunoinhibitors base on TISIDB. **b**–**g** CSF1R had linked with six hub genes base on GEPIA

**Fig. 7:**
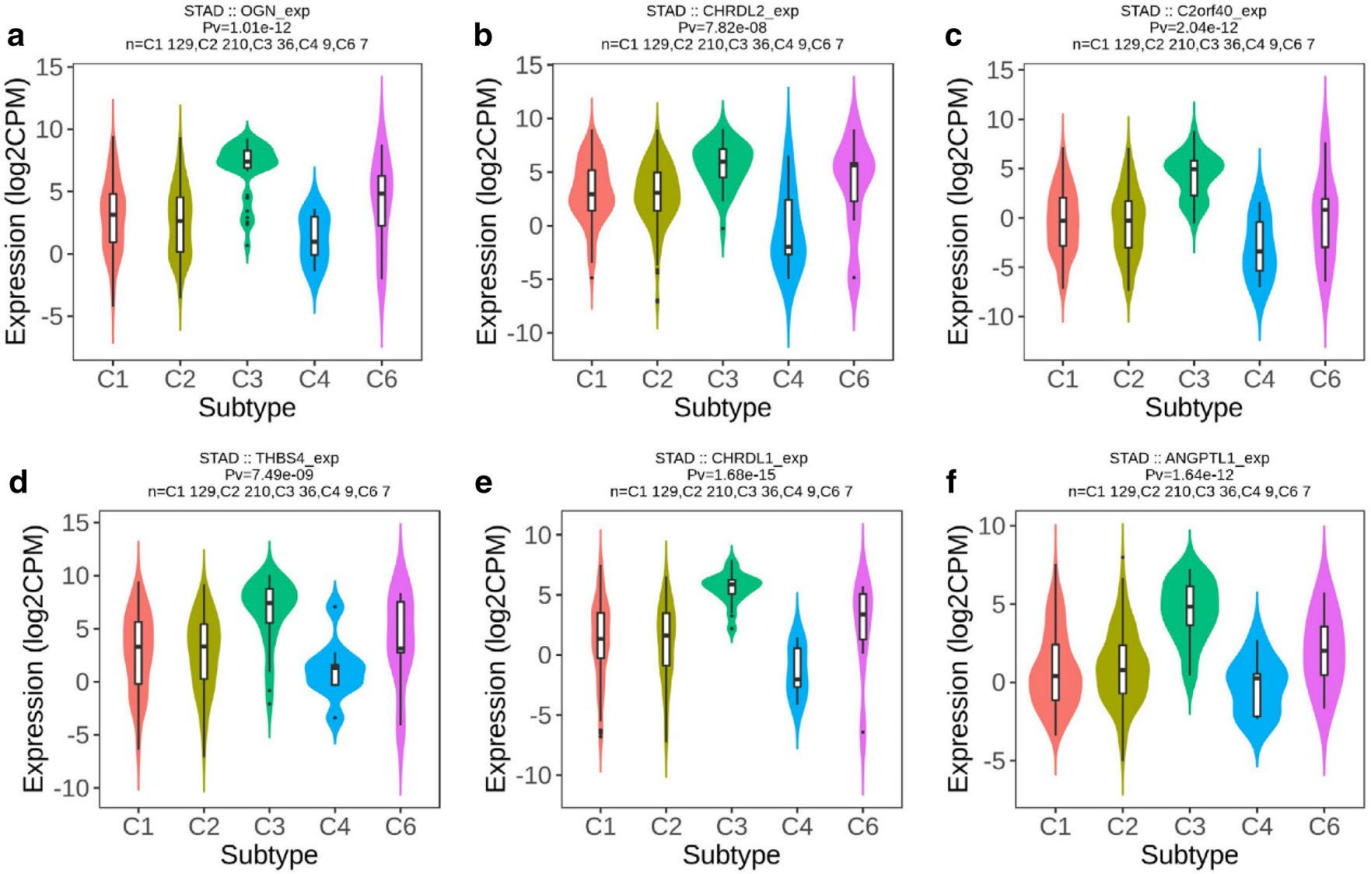
Associations between expression of six hub genes and immune subtypes in GC. OGN (**a**), CHRDL2 (**b**), C2orf40 (**c**), THBS4 (**d**), CHRDL1 (**e**) and ANGPTL1 (**f**) investigated in TISIDB. C1: wound healing; C2: IFN-γ dominant; C3: inflammatory; C4: lymphocyte depleted; C6: TGF-β dominant

To better understand the immune function of these hub genes, we explored the relationship between their expression and immune markers in TISIDB [24]. Tumor immunology analysis exhibited that the expression of these 6 genes was positively correlated with most immunoinhibitors (Fig. 6a) and majority immunostimulators (Supplementary Fig. S4a). Furthermore, we check out the striking different genes verified again in GEPIA, like CSF1R and Cxcl12, and got similar results (Fig. 6b–g, Supplementary Fig. S4b–g). In the end, we search data found that the expression of OGN, C2orf40, CHRDL2, THBS4, CHRDL1, and ANGPTL1, respectively, correlated with different immune subtypes in GC (Fig. 7a–f). Molecular classification of GC benefited patients to gain precisely targeted therapies [25]. Previous studies that separately GC patients into 5 subtypes containing Epstein-Barr virus (EBV)-positive, hypermutated-single-nucleotide variant predominant (HM-SNV), hypermutated enriched for insertion/deletion (HM-indel, which lead to microsatellite instability), chromosomal instability (CIN), and genomically stable (GS) [25, 26]. We then explored the six hub genes expression in various molecular subtypes in GC (Supplementary Fig. S5a–f). In summary, these results implied that these 6 hub genes might potentially govern the conscription and awakening of immune cells in GC.

## Discussion

Chemotherapy, radiotherapy, surgery, immunotherapy, and targeted therapy are effective in the progression of GC in appropriate indications. While the limited OS that no more than 30% in most countries, there are still need specific biomarkers to guide rational using drugs, especially for ICB treatment.

OGN is a member of the small leucine-rich proteoglycan (SLRP) family and its function may vary from different tumors. However, this is the first time that the role of OGN in GC has been reported so far. There was experimental evidence that OGN was upregulated and function as a tumor promoter by inhibiting NF2 expression and triggering mTOR signaling in meningioma [27], but OGN expression reduced and inhibited cell proliferation, invasion, and epithelial to mesenchymal transition through EGFR/Akt pathway in colorectal cancers [28]. While the work model of OGN in GC is not clear and needs to be further explored. Moreover, OGN, as one of the biologically active elements of the vascular extracellular matrix, could be tested in plasma/serum and acted as a biomarker in disease [29, 30]. For instance, serum OGN was an independent risk predictor for patients with chronic kidney disease [29]. This led us to speculate that OGN might serve as a serological marker for the prognosis of GC, which required to be confirmed by extra experiments. Furthermore, OGN also could adjust immune response via mediating immune cell infiltration in cancer, such as OGN expression positively associated with CD8+ T cells recruitment/infiltration by inhibited HIF-1α/ VEGF pathway in colorectal cancer [31]. Here, our results also indicated that OGN was significantly positively correlated with immune cell infiltration in GC, such as M2 macrophages. OGN might be an immune modulator in TME in GC. In a word, our study showed that OGN could be a new useful prognostic biomarker and immune regulator for GC.

CHRDL2, an antagonist of bone morphogenic proteins (BMPs), worked as an oncogene in colorectal cancer [32] and osteosarcoma [33]. CHRDL2 was firstly discovered in 2003 named as BNF-1 (breast tumor novel factor 1) and found its overexpression in breast, lung, and colon tumors detected by PCR in a small sample [34]. While the report of CHRDL2 in the tumor is very limited. Here, we first pointed out that CHRDL2 expression increased in the GC process and its high expression was associated with poor prognosis in GC patients. As a secreted protein, we found its expression associated with immune cell infiltration, especially for M2 macrophage, and immune inhibit molecular markers in GC. Therefore, CHRDL2 might be a novel target of GC therapy.

C2orf40 encodes a protein called esophageal cancer-related gene-4 (ECRG4), which is down-regulated by hypermethylation of its promoter in diverse types of tumors, including hepatocellular carcinoma [35], GC [36], breast cancer [37]. According to current reports, c2orf40 might as a potential tumor suppressor gene in tumors. However, our results exhibited that high expression of ECRG4 was remarkably associated with poor outcomes (HR = 1.95, log-rank P = 6.7e−10), and its expression increased gradually in GC advance. The opposite result may be on account of different levels of evidence or cohort studies [36, 38]. The deep reasons are yet to be further validated. It has been reported that ECRG4 interacted with TLR4 [36]. And TLR4/PI3K/Akt signaling was a vital way to promote M2 polarization of macrophages [10]. Our results exhibited C2orf40/ ECRG4 expression notably related to immune cell infiltration, including M2 macrophages. Thus, it also needs to verify whether ECRG4 is involved in the development of GC by modifying the M2 polarization of macrophages through TLR4.

THBS4 belongs to the thrombospondin protein family, which is a kind of adhesive glycoproteins that mediate cell-to-cell and cell-to-matrix interactions. THBS4 tended to be an oncogene in GC [39], colorectal cancer [40], prostate cancer [41], and hepatocellular carcinoma [42]. As an illustration, inhibited expression of THBS4 could impede the PI3K/Akt signaling and disturb the cancer stem cell (CSC)-like properties in prostate cancer [41]. And THBS4 might as a biomarker for diffuse-type gastric adenocarcinomas [43], and a potential indicator for risk assessment and prognosis prediction of GC according to its polymorphisms [44] and bioinformatics analysis [45]. In addition, THBS4 is a secreted extracellular matrix protein that has been reported to mediate angiogenesis, adhesion, migration, and proliferation responding to TGF-β signaling [46]. Our results suggested THBS4 expression was associated with TGFβ in GC. Whether THBS4 responds to TGFβ signaling pathway in GC and participates in the progression of GC remains to be verified experimentally. Our study found that the expression of THBS4 was notably increased following the advanced procession of GC and TGFβ pathways might also facilitate THBS4 secretion to promoting GC. THBS4 also positively correlated with others immunoinhibitors and majority immunostimulators. The important role of THBS4 in GC still needs more evidence to prove.

CHRDL1 is a paralog of CHRDL2, which has only been reported in two papers in GC. One paper reported that its expression associated with CLIP4 DNA methylation and CHRDL1 might be a prognostic signature gene [47]. Another reported low expression of CHRDL1, as an antagonist of bone morphogenetic protein 4 (BMP4), might promote GC cell proliferation and migration by BMP receptor II [48]. However, our results revealed that high expression of CHRDL1 with poor OS in GC by detecting in two datasets, and CHRDL1 expression was associated with tumor stages, immune cell infiltration, immunoinhibitors, and immunostimulators.

Many papers showed ANGPTL1 acted as a tumor suppressor by inhibiting angiogenesis, cancer metastasis, cancer stemness, and repressing sorafenib resistance in treatment [49, 50]. While its role in GC still not clear [51]. Our results revealed that ANGPTL1 might be a potential target in GC, according to its high expression related to poor OS and gradually increased with tumor stages as well as the feasible relationship with immune cells and biomarkers in TME.

## Conclusions

Here, we searched a series of databases to find out the most relevant molecular that their expression closely related to the survival of GC. And functional enrichment analysis of survival-related CMS genes was performed. These genes are involved in many cancers, including GC, and cancer processes, such as angiogenesis. Then, we picked 6 genes that could be secreted in the TME and enhanced in immune cells. Further survival analysis demonstrated that high expression of OGN, CHRDL2, C2orf40, THBS4, CHRDL1, and ANGPTL1, respectively, was significantly associated with poor OS in GC patients. OGN and CHRDL2 were the firstly reported in GC. Moreover, the expression of these 6 genes prominently increased with the tumor pathological stage. In addition, we investigated the pathways of those hub genes by enrichment analysis. Ultimately, immune infiltration analysis displayed that those hub genes expression positively with CD8+ T Cell, M2 macrophage, most immunoinhibitors, and majority immunostimulators. These processes are closely relative to tumor growth and metastasis. Therefore, high expression of 6 hub genes alone or synergistically resulted in a poor prognosis in GC patients. More experiments are still needed to verify these findings.

## Materials and methods

### COMSUC

COMSUC [12] (http://comsuc.bioinforai.tech/home) is used to identify Consensus Molecular Subtypes (CMS) by integrating multiple clustering results based on multiple platforms, multiple omics data, and multiple methods. In this study, we integrated clustering results of GC data from TCGA into two groups by three algorithms based on K-means, hierarchical clustering (Hclust), non-negative matrix factorization (NMF).

### Metascape

Metascape [13] (http://metascape.org/gp/index.html#/main/step1) is a web server designed to provide an extensive gene list annotation and analysis resource for users. Enrichment analysis was the essential part of Metascape. Here, we analyzed the prominent changed genes of CMS1 for enrichment analysis by custom analysis.

### Kaplan–Meier plotter

Kaplan–Meier plotter [18] (http://kmplot.com/analysis/) is a platform utilized to discover and validate the survival biomarkers of four cancers, including gastric cancer. To analyze the prognostic value of OGN, CHRDL2, C2orf40, THBS4, CHRDL1, and ANGPTL1, the cohorts were divided into high- and low- groups through their expression. In this study, the overall survival of 6 hub genes was compared in GC. And the hazard ratios (HRs, with 95% confidence intervals) and log-rank P-values (< 0.05 as significant difference) were counted.

### GEPIA

Gene Expression Profiling Interactive Analysis [22] (GEPIA, http://gepia.cancer-pku.cn/) uses a standard processing approach to examine genes expression by RNA sequencing data for 8587 normal samples and 9736 tumors from GTEx and TCGA projects. Here, GEPIA was used to investigate the six hub genes expression correlation for GC tumor stages from TCGA data. In addition, we valued the correlation between the expression of CSF1R/CXCL12 and six hub genes base on GEPIA.

### TIMER

TIMER [23] (https://cistrome.shinyapps.io/timer/) is a web tool used to investigate immune cell infiltration in diverse cancers, including GC. It provides data to evaluate the associations between expression levels of selected genes and infiltrating immune cells, such as B cells, CD4+ T cells, CD8+ T cells, neutrophils, macrophages, and dendritic cells. In our study, we estimated the correlation of expression of OGN, CHRDL2, C2orf40, THBS4, CHRDL1, and ANGPTL1 with immune cell infiltration.

### TISIDB

TISIDB [24] (http://cis.hku.hk/TISIDB/index.php) is an online web for assessing tumor-related gene and immune system interaction. In our study, we used TISIDB to find out the correlation between the expression of six hub genes and the abundance of immunomodulators. And distribution of six hub genes expression across immune and molecular subtypes in GC. The correlations between six hub genes and the immune system were measured by Spearman’s test.

## Supplementary Information

Supplementary file 1. (DOCX 3417 KB)

## Data Availability

All data generated or analyzed during this study are included in this article.

## Abbreviations

GC: Gastric cancer
ICB: Immune checkpoint blockade
TME: Tumor microenvironment
OS: Overall survival
CMS: Consensus molecular subtypes
TFs: Transcription factors
SLRP: Small leucine-rich proteoglycan
BMPs: Bone morphogenic proteins
BNF-1: Breast tumor novel factor 1
ECRG4: Esophageal cancer-related gene-4
CSC: Cancer stem cell
