# Supplementary material for: Identification hub genes of consensus molecular subtype correlation with immune infiltration and predict prognosis in gastric cancer
Source: Discov Oncol. 2021 Oct 16;12:41. doi: 10.1007/s12672-021-00434-5 (PMC8777542; doi:10.1007/s12672-021-00434-5)
Supplement: Supplementary file 1 — (DOCX 3417 KB) [file 12672_2021_434_MOESM1_ESM.docx]

Supplementary Figures S1-S5 and Figure legends


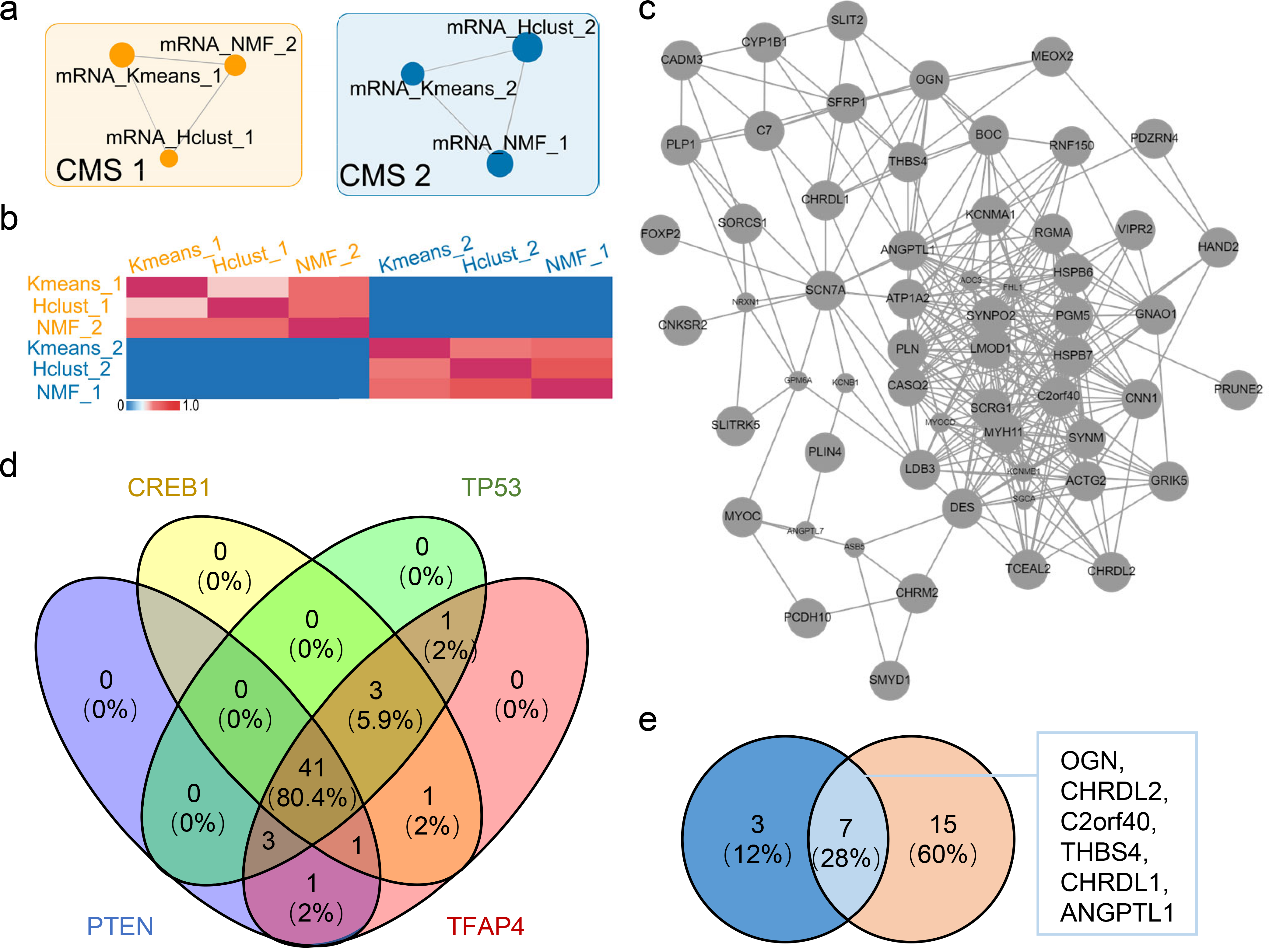


**Fig. S1 Selecting the hub genes significantly correlated with the survival of GC patients.**

**a** Network of GC CMS by K-means, hierarchical clustering (Hclust), non-negative matrix factorization (NMF). **b** Heatmap of GC by two groups of CMS across three clustering methods. **c** PPI network of CMS selected genes in GENEMANIA database. **d** Venn diagram showed the number of those selected 52 genes regulated by TFAP4, TP53, CREB1, PTEN predicted in Knock TF. **e** Venn diagram displayed genes that encode secreted proteins and enriched in immune cells.

**
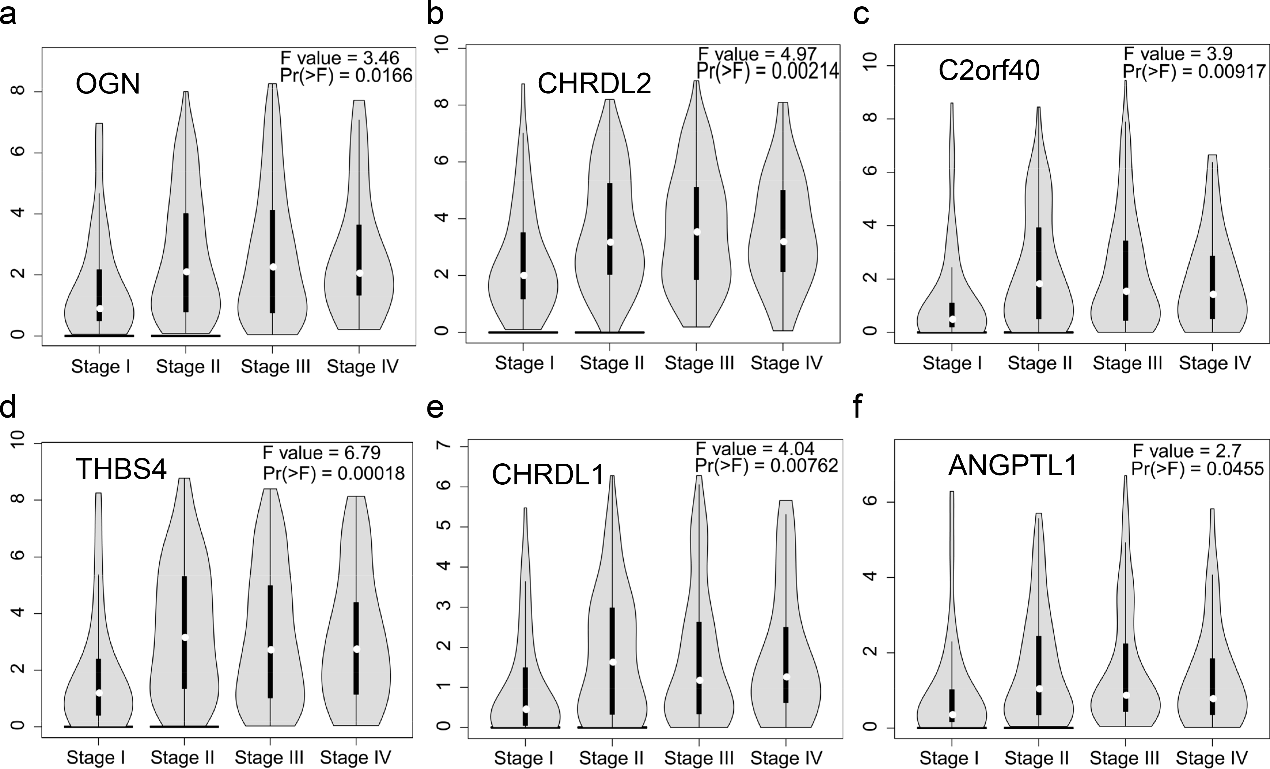
**

**Fig. S2 Relationships between mRNA levels of** **six hub genes and** **tumor stages.**

mRNA expression of OGN, CHRDL2, C2orf40, THBS4, CHRDL1, and ANGPTL1 was significantly correlated with tumor stages based on GEPIA.


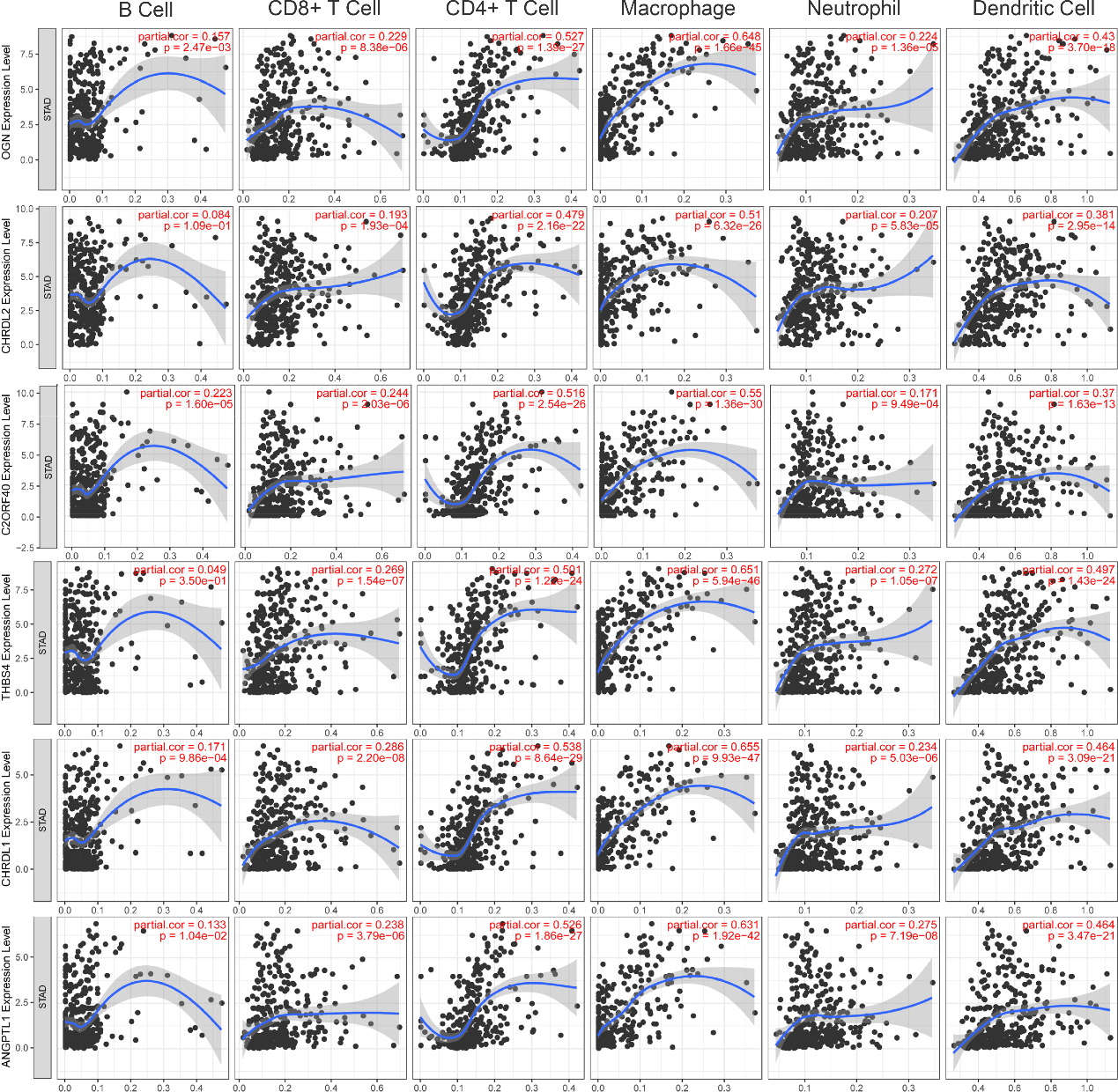


**Fig. S3 Correlations of six hub genes with immune cell infiltration level in GC.**

OGN, CHRDL2, C2orf40, THBS4, CHRDL1, and ANGPTL1 all had significant positive correlations with infiltrating levels of CD8+ T cells, CD4+ T cells, macrophages, neutrophils, dendritic cells in GC.


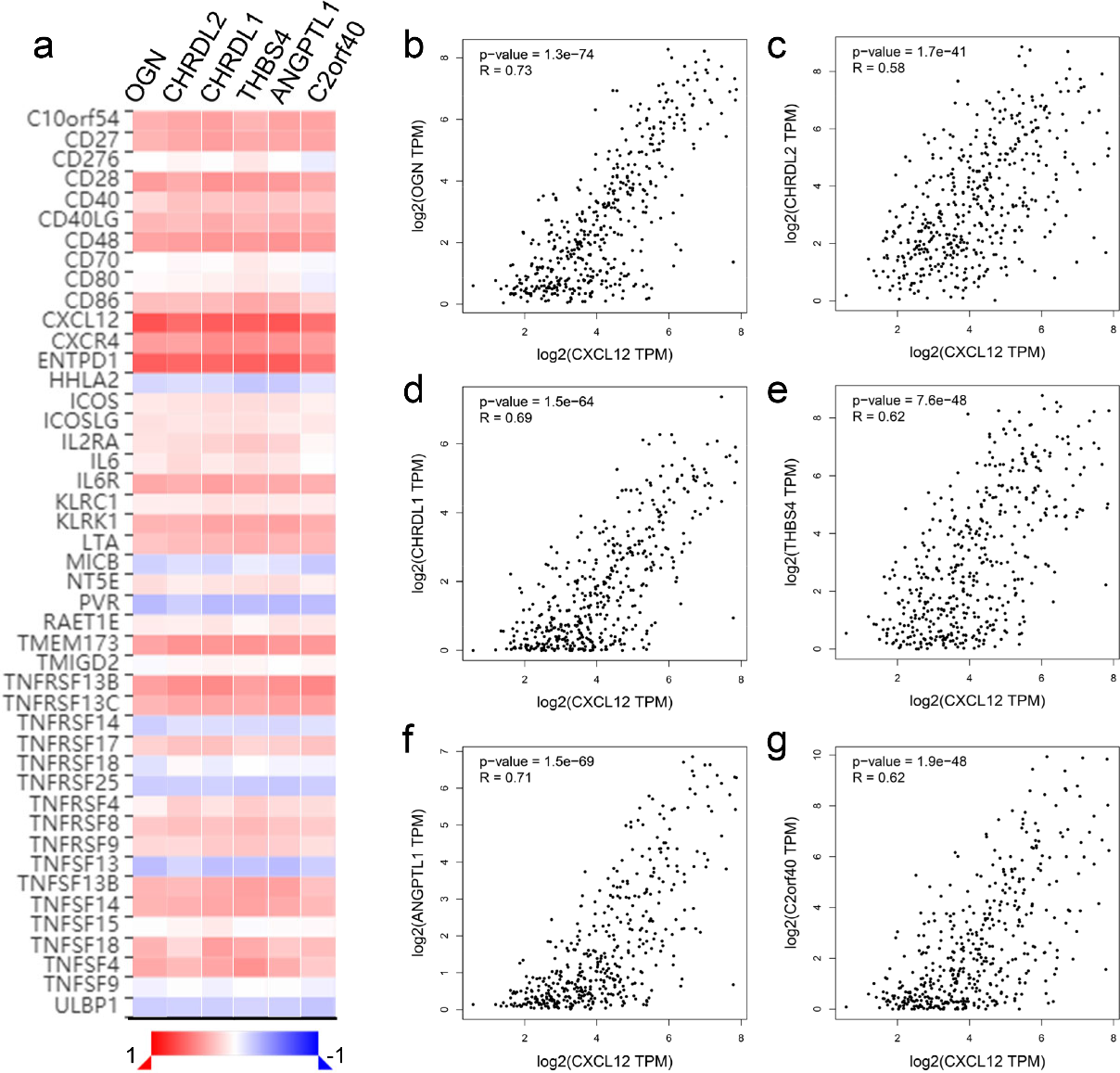


**Fig. S4** Tumor immunology analysis exhibited the expression of six hub genes' relationship with immunostimulators base on TISIDB (a**)** and GEPIA (b-g**).**


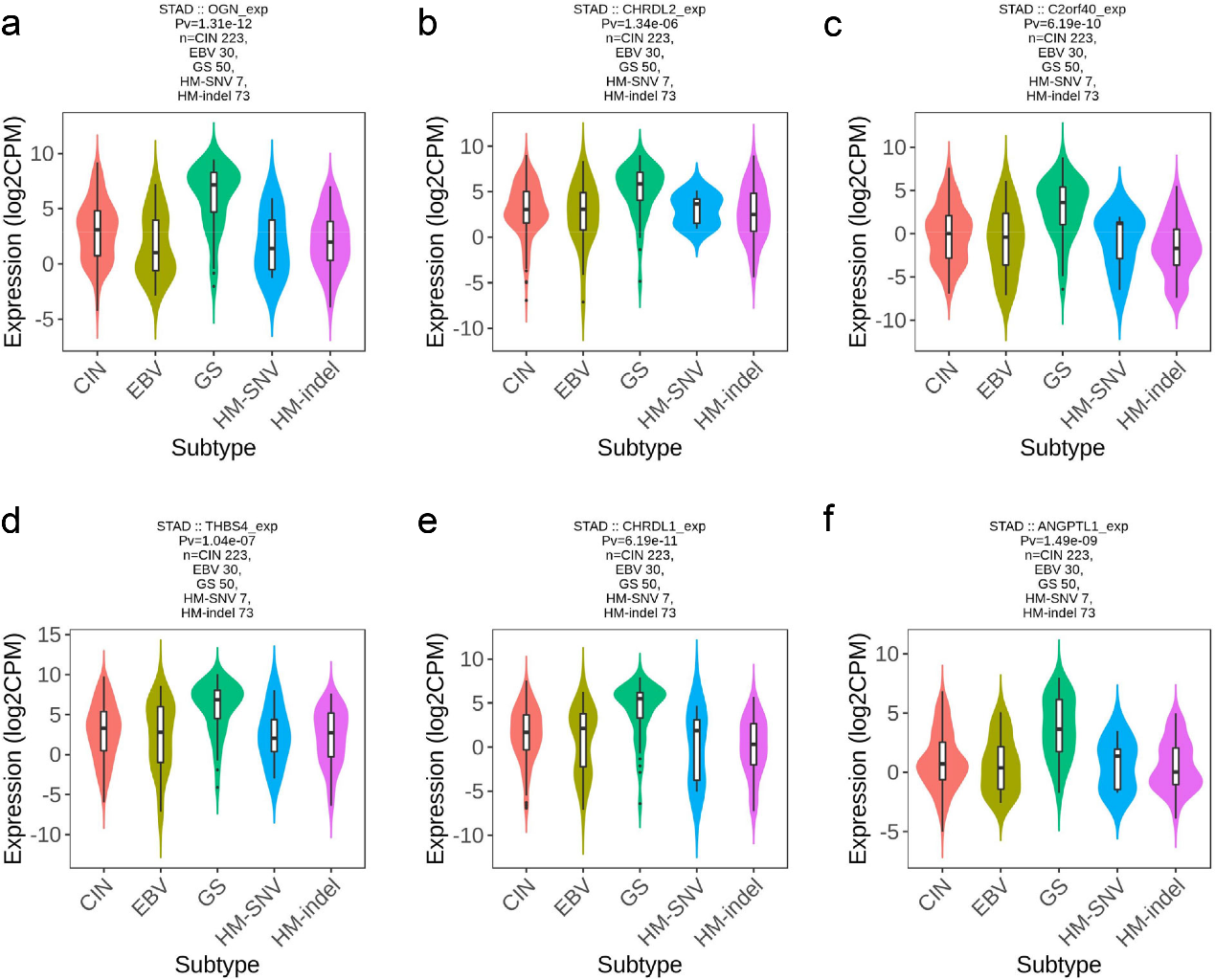


**Fig. S5 Correlations between expression of six hub genes and molecular subtypes in GC.**

OGN (a), CHRDL2 (b), C2orf40 (c), THBS4 (d), CHRDL1(e) and ANGPTL1 (f) analyzed in TISIDB. CIN: chromosomal instable; EBV: Epstein–Barr virus–positive; GS: genomically stable; HM-SNV: hypermutated with elevated single nucleotide variation; HM-indel: hypermutated enriched for insertion/deletion.
